# Supplementary material for: Does Seasonal Influenza Related Hospital Occupancy Surge Impact Hospital Staff Sickness Presenteeism and Productivity Costs?
Source: Int J Environ Res Public Health. 2022 Jan 11;19(2):769. doi: 10.3390/ijerph19020769 (PMC8775749; doi:10.3390/ijerph19020769)
Supplement: Supplementary file 1 [file ijerph-19-00769-s001.zip › ijerph-1476266-supplementary.pdf]

Supplementary Table S1. Conventional and multilevel logistic regression results on presenteeism frequency amongst nurses for individual and hospital level characteristics

	Generalized estimating equation (GEE) regression Adjusted OR (95% CI) [a,b,c]	Conventional Logistic Regression Adjusted OR (95% CI) [b]
**Intercept**	3.03** (1.96, 4.68)	3.03** (1.94, 4.72)
**Hospital-level risk factors**		
Occupancy Z score	1.19** (1.05, 1.35)	1.18 (0.98, 1.42)
Hospital		
H1	-	1.19 (0.84, 1.67)
H2	-	0.83 (0.61, 1.14)
H3	-	1
**Nurse Characteristics covariates**		
**Age Group**		
≤30	1	1
31-40	1.82** (1.37, 2.41)	1.84** (1.39, 2.44)
41-50	1.46* (1.09, 1.95)	1.43* (1.08, 1.91)
≥51	1.50* (1.02, 2.21)	1.48* (1.01, 2.16)
**Rank**		
Junior staff (EN)	1.07 (0.67, 1.71)	1.08 (0.68, 1.70)
Junior staff (RN)	1	1
Middle management (APN/NC)	0.68** (0.51, 0.89)	0.68** (0.52, 0.89)
**Working Schedule**		
Regular schedule (9am-5pm)	1.42 (0.98, 2.05)	1.44 (1.00, 2.07)
Shift schedule	1	1
**Main working location**		
A&E	0.99 (0.66, 1.48)	1.00 (0.67, 1.50)
Medicine [d]	1	1
Surgery [e]	1.04 (0.85, 1.29)	1.02 (0.83, 1.25)
**Health**		
Poor (score: 1,2)	1	1
Normal (score 3)	0.45** (0.35, 0.58)	0.45** (0.35, 0.58)
Good (score: 4,5)	0.26** (0.20, 0.33)	0.26** (0.20, 0.33)
**Sick leave**	1.08* (1.00, 1.17)	1.12* (1.03, 1.23)

* p<0.050, **p<0.001

[a] Nurses who are in senior management or mainly work at GOPC/SOPC or other departments (administration, management, residential care, public health, rehabilitation, occupational health, community nursing, mental health, psychiatry, addiction treatment and others) were excluded in the analysis so to measure the effect of workload on presenteeism amongst inpatient ward nurses only

[b] model adjusted for hospital, age, rank, shift, health, primary working function and sick leave

[c] Corrected for small-sample bias

[d] Medicine – includes medicine, geriatrics, pediatrics and intensive care unit (ICU)

[e] Surgery – includes surgery, obstetrics, gynecology and operation theatre

Note: GOPC: General out-patient clinics; SOPC: Specialist Outpatient Clinic; OR: Odds ratio; CI: confidence interval.
